# High Concentrations of Genistein Decrease Cell Viability Depending on Oxidative Stress and Inflammation in Colon Cancer Cell Lines

**DOI:** 10.3390/ijms23147526

**Published:** 2022-07-07

**Authors:** Marina Alorda-Clara, Margalida Torrens-Mas, Pere Miquel Morla-Barcelo, Pilar Roca, Jorge Sastre-Serra, Daniel Gabriel Pons, Jordi Oliver

**Affiliations:** 1 Grupo Multidisciplinar de Oncología Traslacional, Institut Universitari d’Investigació en Ciències de la Salut (IUNICS), Universitat de les Illes Balears, E-07122 Palma de Mallorca, Illes Balears, Spain; marina.alorda@uib.es (M.A.-C.); margalida.torrens@ssib.es (M.T.-M.); pere.morla@uib.es (P.M.M.-B.); pilar.roca@uib.es (P.R.); jorge.sastre@uib.es (J.S.-S.); jordi.oliver@uib.es (J.O.); 2 Instituto de Investigación Sanitaria Illes Balears (IdISBa), Hospital Universitario Son Espases, Edificio S, E-07120 Palma de Mallorca, Illes Balears, Spain; 3 Translational Research in Aging and Longevity (TRIAL) Group, Instituto de Investigación Sanitaria Illes Balears (IdISBa), E-07120 Palma de Mallorca, Illes Balears, Spain; 4 Ciber Fisiopatología Obesidad y Nutrición (CB06/03), Instituto Salud Carlos III, E-28029 Madrid, Madrid, Spain

**Keywords:** colorectal cancer, genistein, cell viability, hydrogen peroxide production, mitochondrial biogenesis, inflammation

## Abstract

Genistein could play a crucial role in modulating three closely linked physiological processes altered during cancer: oxidative stress, mitochondrial biogenesis, and inflammation. However, genistein’s role in colorectal cancer remains unclear. We aimed to determine genistein’s effects in two colon cancer cells: HT29 and SW620, primary and metastatic cancer cells, respectively. After genistein treatment for 48 h, cell viability and hydrogen peroxide (H_2_O_2_) production were studied. The cell cycle was studied by flow cytometry, mRNA and protein levels were analyzed by RT-qPCR and Western blot, respectively, and finally, cytoskeleton remodeling and NF-κB translocation were determined by confocal microscopy. Genistein 100 µM decreased cell viability and produced G_2_/M arrest, increased H_2_O_2_, and produced filopodia in SW620 cells. In HT29 cells, genistein produced an increase of cell death, H_2_O_2_ production, and in the number of stress fibers. In HT29 cells, mitochondrial biogenesis was increased, however, in SW620 cells, it was decreased. Finally, the expression of inflammation-related genes increased in both cell lines, being greater in SW620 cells, where NF-κB translocation to the nucleus was higher. These results indicate that high concentrations of genistein could increase oxidative stress and inflammation in colon cancer cells and, ultimately, decrease cell viability.

## 1. Introduction

Genistein (GEN) is a phytoestrogen that belongs to the isoflavones class and is found in soybeans [1]. Asian countries have a higher intake of this isoflavone than European countries because of the level of their soy-derived products consumption [2]. It is known that tumors can be affected by GEN, but the goodness of the effect is still unknown [3]. Colorectal cancer (CRC) is the third-most common cancer and the second-most common cause of cancer death worldwide [4]. Until now, Asia had the lowest CRC incidence, but nowadays it has increased [5]. This increment in the number of CRC cases in Asia can be due to changes in diet, which now is more westernized [6]. CRC is one of the most affected cancers by GEN, as GEN promotes apoptosis, cell cycle arrest, and a decrease in cell proliferation and metastasis in this cancer, but the mechanism by how GEN affects CRC remains unclear [1,3,7].

The chemopreventive activity of GEN can modulate different physiological processes during cancer, such as oxidative stress [1], cancer cells bioenergetics [8], and inflammation [9], which are important in different phases of CRC [10,11,12].

GEN has a dual effect in front of oxidative stress. It is well known that GEN acts as an antioxidant during oxidative stress, increasing antioxidant enzymes’ expression [1], but GEN can also act as a pro-oxidant when it is present at high concentrations [13,14,15]. Oxidative stress is the result of a disbalance between reactive oxygen species (ROS) production and antioxidant defenses [16]. Mitochondria are the major producers of ROS in cells during an electronic transport chain [17,18] and form free radicals by the reduction of molecular oxygen, forming superoxide and hydroxyl radicals [17], which are detoxified by the antioxidant enzymes (superoxide dismutases, peroxidases, and catalase) [17,18]. Oxidative stress is involved in the initiation and progression of cancer due to the capacity of ROS to increase DNA mutations, oxidative damage to macromolecules, genomic instability, and cellular proliferation [10]. Furthermore, moderate levels of ROS can decrease apoptosis, interrupt cell–cell communication, and modify second messenger systems [10]. Finally, ROS can activate tumor suppressors in a reversible form [16].

ROS production is closely related to mitochondrial biogenesis, which is a complex process where the nuclear and mitochondrial genome must be highly regulated, since the nuclear genome codifies proteins which control the transcription and replication of mtDNA that, in turn, codifies some mitochondrial proteins such as electron transport chain proteins [19]. This process involves both the replication and transcription of mtDNA [19], and it is a highly regulated pathway [20]. Mitochondrial biogenesis and functionality have always been believed to be compromised in cancer development [11]. The role of mitochondria in metastatic CRC has not been well established yet, but some studies suggested that cells from more advanced stages have a higher oxidative phenotype compared to cells from earlier stages [21]. Finally, GEN could be playing a role in cancer cells’ bioenergetics. However, the role of GEN on mitochondrial biogenesis is not well established [8].

In the same way, colorectal tumors suffer an increase of proinflammatory cytokines’ expression and a constitutive activation of transcription factors related to inflammatory pathways [22]. These changes in inflammation can be done, at least in part, by mitochondria, since ROS can directly activate inflammatory pathways [23] and inflammasomes [24], which is a multiprotein complex that releases proinflammatory cytokines [25]. Furthermore, when mitochondrial damage is present, mitochondrial DNA is released into the cytosol, where it can activate the inflammasomes [25]. The established cytokine network allows for survival, growth, proliferation, differentiation, immune cells’ activation, and tumoral and stromal cells’ migration [12]. Inflammation is achieved with the release of cytokines, chemokines, and the activation of pathways related to inflammation participates in all phases of CRC, as it allows tumoral growth and it is associated to angiogenesis, the epithelial–mesenchymal transition, and metastasis [12].

Our aim was to investigate the effects of genistein treatment in oxidative stress, mitochondrial biogenesis, and inflammatory parameters in two colon cancer cell lines: HT29, a primary and moderately differentiated cell line, and SW620, a metastatic and poorly differentiated cell line. For this goal, cell viability, ROS production, the cellular cycle, gene and/or protein expression levels of antioxidant enzymes, mitochondrial biogenesis regulators and inflammation-related genes, the nuclear translocation of nuclear factor kappa B (NF-κB), and actin cytoskeleton remodeling were studied.

## 2. Results

### 2.1. High Concentrations of Genistein Decreased Cell Viability

**Figure 1:**
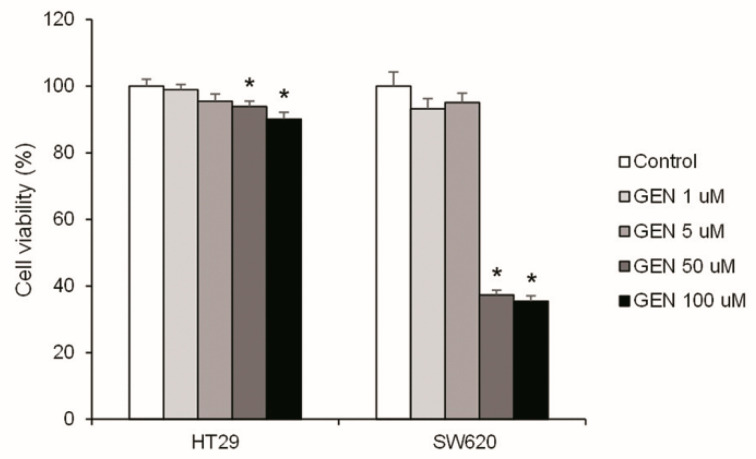
Effects of different concentrations of genistein on cell viability in HT29 and SW620 cells, determined with Hoechst 33342 assay. The value of control cells (DMSO-treated cells) was set at 100%. The coordinate values represent cell viability (%) and the measurements were made with genistein 1, 5, 50, and 100 μM or 0.1% DMSO (control cells) treatment for 48 h. GEN, genistein. * Significant difference between control cells and genistein-treated cells (Student’s *t*-test; *p* < 0.05, *n* = 6).

Cell survival is key in cancer progression; consequently, the effect of GEN in cell viability was studied. Figure 1 shows the cell viability analysis after 48 h of treatment with increasing concentrations of GEN (1, 5, 50, and 100 μM). Only the highest concentrations of GEN (50 and 100 μM) caused a statistically significant reduction in cell viability. This decrease was more pronounced in SW620 cells (−63% and −65%, respectively) than in HT29 cells (−6% and −10%, respectively).

### 2.2. GEN Produced Cell Cycle Arrest and Apoptosis

**Figure 2:**
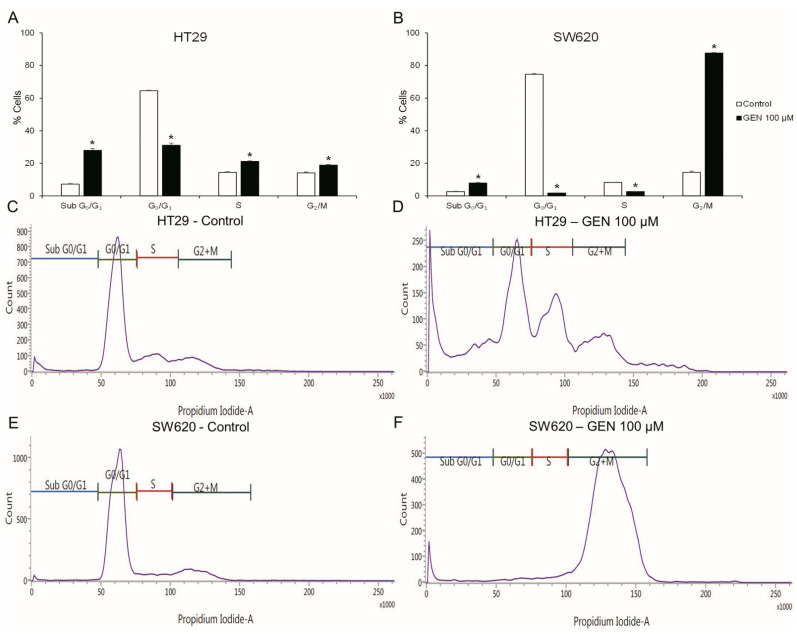
Effects of genistein on cell cycle in (**A**) HT29 cells and (**B**) SW620 cells, determined by flow cytometry. Representative event count and propidium iodide fluorescence in HT29 cells (**C**,**E**) and SW620 cells (**D**,**F**). The measurements were made with 0.1% DMSO (control cells, (**C**,**D**)) or genistein 100 μM (**E**,**F**) treatment for 48 h. Values are expressed as mean ± SEM. GEN, genistein. * Significant difference between control cells and genistein-treated cells (Student’s *t*-test; *p* < 0.05, *n* = 6).

The cell cycle (Figure 2) was studied with flow cytometry after 48 h of GEN 100 μM treatment. HT29 cells (Figure 2A) suffered a decrease in the G_0_/G_1_ phase (−52%), but suffered an increase in the Sub G_0_/G_1_, S and G_2_/M phases (+288%, +46%, and +33%, respectively). SW620 cells (Figure 2B) presented a decrease in the G_0_/G_1_ and S phases (−97% and −68%, respectively) and an increase in the Sub G_0_/G_1_ and G_2_/M phases (+199% and +504%, respectively).

### 2.3. High Concentrations of Genistein Increased H_2_O_2_ Production

**Figure 3:**
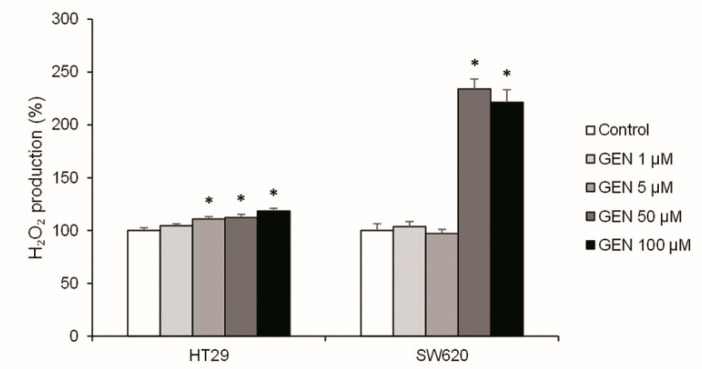
Effects of different concentrations of genistein on H_2_O_2_ production determined with Amplex^®^ Red Hydrogen Peroxide/Peroxidase Assay in HT29 and SW620 cells. The coordinate values represent H_2_O_2_ production (%) and values are expressed as mean ± SEM and are normalized as percentage of control values (DMSO-treated cells). The measurements were made with genistein 1, 5, 50, and 100 μM or 0.1% DMSO (control cells) treatment for 48 h. GEN, genistein. * Significant difference between control cells and genistein-treated cells (Student’s *t*-test; *p* < 0.05, *n* = 6).

To know the origin of cell death, the oxidative stress status was studied. The H_2_O_2_ production was analyzed after 48 h of GEN treatment (Figure 3). HT29 cells presented a weak increase of the H_2_O_2_ production in a dose-dependent manner, reaching +18% with the highest concentration. However, in SW620, the results showed an accentuated increase in H_2_O_2_ production when GEN concentrations were higher (+120% approximately at 50 and 100 μM), without significant changes with the lower concentrations.

### 2.4. GEN Modulated the Expression of Different Antioxidant Enzymes

**Figure 4:**
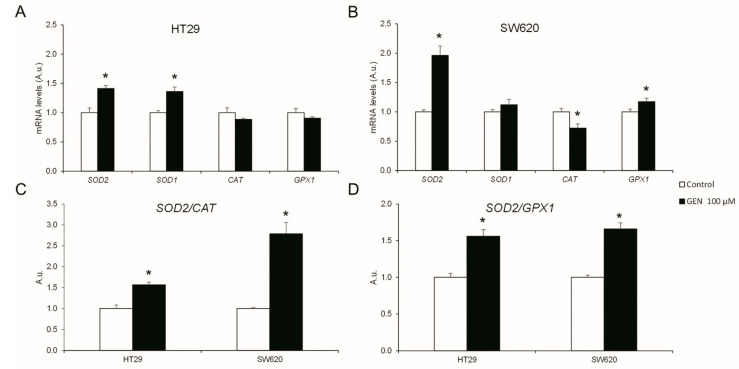
Effects of genistein on antioxidant enzymes’ mRNA expression levels (*SOD2, SOD1, CAT*, and *GPX1*) in (**A**) HT29 cells and (**B**) SW620 cells determined by real-time PCR. Effects of genistein on mRNA expression levels ratios of (**C**) *SOD2/CAT* and (**D**) *SOD2/GPX1* genes determined by real-time PCR. The measurements were made with genistein 100 μM or 0.1% DMSO (control cells) treatment for 48 h. The coordinate values represent mRNA levels (arbitrary units). Values are expressed as mean ± SEM and control values were set at 1.00. GEN, genistein; A.u., arbitrary units. * Significant difference between control cells and genistein-treated cells (Student’s *t*-test; *p* < 0.05, *n* = 6).

To understand the changes in H_2_O_2_ production, the mRNA expression levels of different antioxidant enzymes were studied after 48 h of GEN 100 µM treatment. HT29 cells (Figure 4A) suffered a statistically significant increase in *SOD2* (+40%) and *SOD1* (+36%) mRNA expression levels after GEN treatment. Moreover, in SW620 cells (Figure 4B), GEN treatment caused a statistically significant increase in the expression levels of *SOD2* (+96%) and *GPX1* (+17%), in addition to a statistically significant decreased expression level of *CAT* (−28%). Furthermore, the changes in the *SOD2/CAT* (Figure 4C) and *SOD2/GPX1* (Figure 4D) ratios were evaluated, since *SOD2* is the mitochondrial dismutase. HT29 cells showed a statistically significant increase in both ratios (+56% in both ratios), as well as SW620 cells (+178% and +66%, respectively).

**Figure 5:**
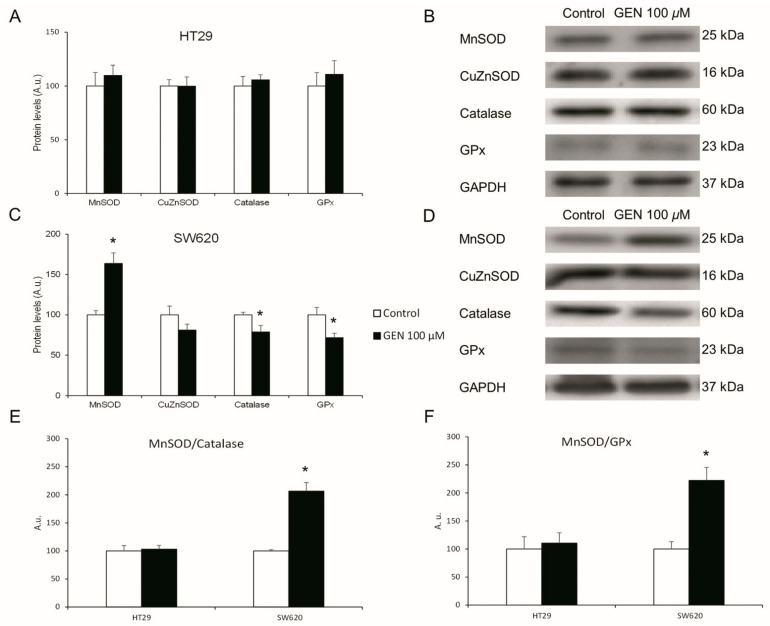
Effects of genistein on protein expression levels. Antioxidant enzymes (MnSOD, CuZnSOD, Catalase, GPx) in (**A**) HT29 cells and (**C**) SW620 cells determined by Western blot. Western blot representative bands in (**B**) HT29 cells and (**D**) SW620 cells. Effects of genistein on protein expression level ratios of (**E**) MnSOD/Catalase and (**F**) MnSOD/GPx determined by Western blot. The measurements were made with genistein 100 μM or 0.1% DMSO (control cells) treatment for 48 h. Values are expressed as mean ± SEM and control values (DMSO-treated cells) were set at 100. GEN, genistein; A.u., arbitrary units. * Significant difference between control cells and genistein-treated cells (Student’s *t*-test; *p*< 0.05, *n* = 6).

SW620 cell line gene expression changes were more pronounced than in HT29 cells. In consequence, the protein expression levels of the same antioxidant enzymes were studied in both cell lines after 48 h of GEN 100 µM treatment. HT29 cells (Figure 5A) showed no changes in their protein expression levels of antioxidant enzymes after GEN treatment. SW620 cells (Figure 5C) showed a statistically significant increase in MnSOD (+64%) protein expression levels, as well as a statistically significant decrease in Catalase (−21%) and GPx (−29%) protein expression levels. Furthermore, the changes in the MnSOD/Catalase (Figure 5E) and MnSOD/GPx (Figure 5F) ratios were evaluated, and SW620 showed a statistically significant increase in both ratios (+106% and +122%, respectively). Figure 5B,D show representative bands in HT29 and SW620, respectively, of the GEN effects on MnSOD, CuZnSOD, Catalase, GPx, and GAPDH expression levels.

### 2.5. GEN Affected Actin Cytoskeleton

**Figure 6:**
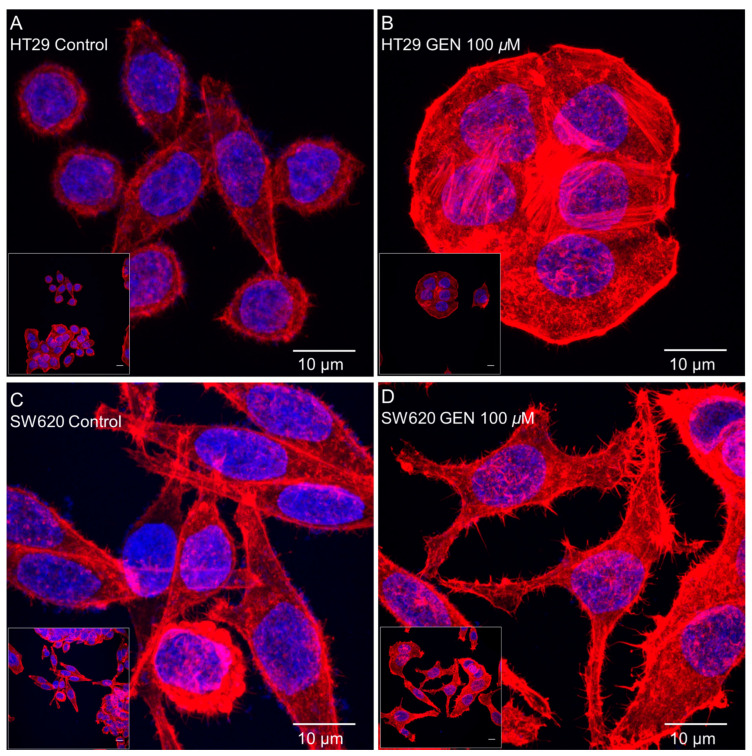
Effects of genistein on actin cytoskeleton in (**A**,**B**) HT29 cells and (**C,D**) SW620 cells, determined by Phalloidin staining. The measurements were made with 0.1% DMSO (control cells, (**A,C**)) or genistein 100 μM (**B,D**) treatment for 48 h. The fluorescence was monitored with a Leica TCS-SPE Confocal Microscope, using 63× immersion oil (147 N.A.) objective lens. Scale bar 10 μm. White square in the bottom left corner shows the whole field without zoom and the image in the center shows a zoom from the white square image. Nucleus and actin cytoskeleton are represented in the picture in blue and red, respectively.

Actin cytoskeleton remodeling was studied with phalloidin staining after 48 h of GEN 100 μM treatment. Figure 6 shows confocal microscopy images taken after phalloidin and DAPI (for the nucleus staining) incubation, where an increase in the number of stress fibers in the HT29 cell line (Figure 6B) and an increase in the number of filopodia in SW620 cells (Figure 6D) after GEN treatment can be observed.

### 2.6. GEN Modified the Expression of Different Mitochondrial Biogenesis Genes

**Figure 7:**
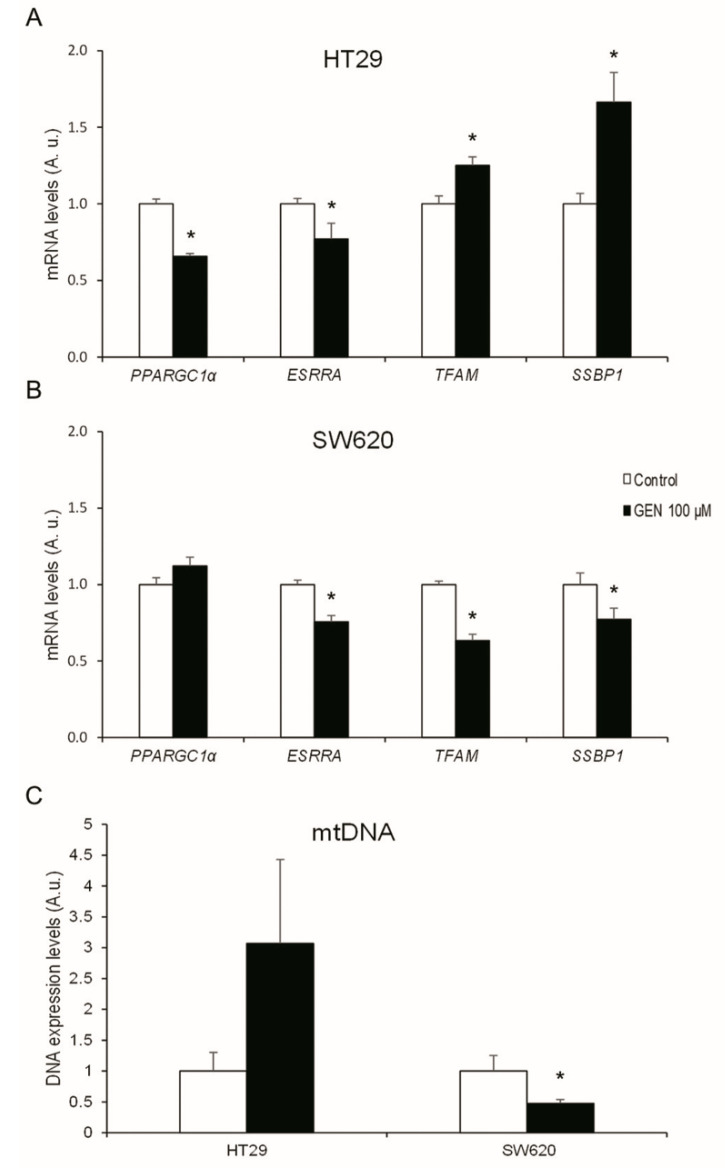
Effects of genistein on mRNA expression levels of mitochondrial biogenesis-related genes (*PPARGC1α, ESRRA, TFAM*, and *SSBP1*) in (**A**) HT29 cells and (**B**) SW620 cells, determined by real-time PCR. Effects of genistein on DNA expression levels of mitochondrial DNA in (**C**) HT29 and SW620 cells determined by real-time PCR. The measurements were made with genistein 100 μM or 0.1% DMSO (control cells) treatment for 48 h. The coordinate values represent mRNA levels (arbitrary units). Values are expressed as mean ± SEM and control values (DMSO-treated cells) were set at 1.00. GEN, genistein; A.u., arbitrary units. * Significant difference between control cells and genistein-treated cells (Student’s *t*-test; *p* < 0.05, *n* = 6).

To understand the changes in the mitochondrial biogenesis regulations, mRNA expression levels of different mitochondrial regulatory genes were studied after 48 h of GEN 100 μM treatment. HT29 cells (Figure 7A) suffered a statistically significant decrease in *PPARGC1α* and *ESRRA* (−35% and −23%, respectively) after GEN treatment, while *TFAM* and *SSBP1* suffered a statistically significant increase (+25% and +66%, respectively). In contrast, SW620 cells (Figure 7B) did not suffer changes in *PPARGC1α*, but *ESRRA*, *TFAM*, and *SSBP1* suffered a statistically significant decrease (−25%, −37%, and −23%, respectively). Furthermore, mitochondrial DNA expression levels (Figure 7C) were also studied after 48 h of GEN 100 μM treatment, presenting a decrease (−53%) in SW620 cells after GEN treatment.

### 2.7. GEN Increased the Inflammatory Status

**Figure 8:**
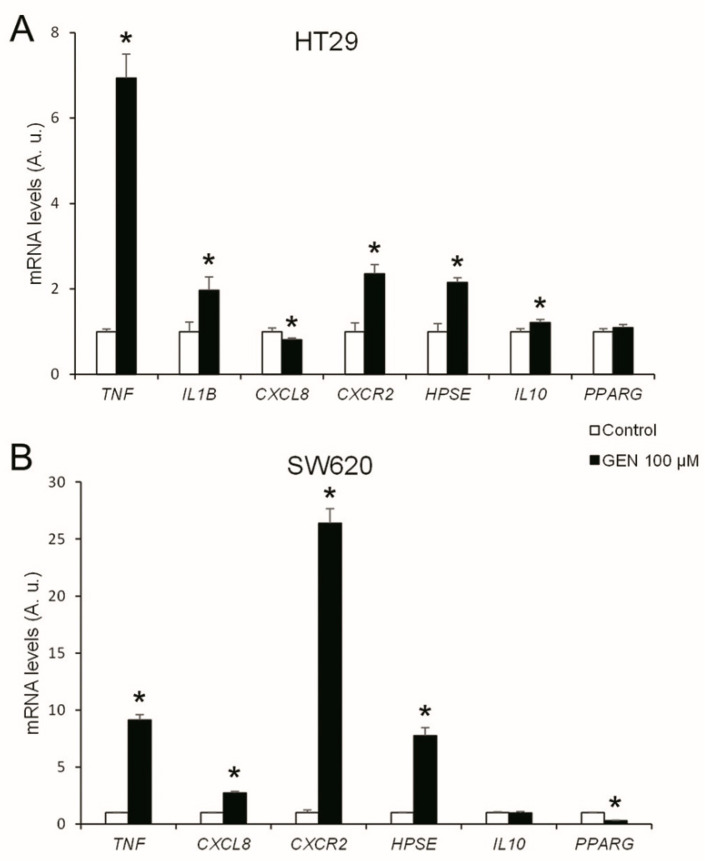
Effects of genistein on mRNA expression levels of inflammation-related genes (*TNF, IL1B, CXCL8, CXCR2, HPSE, IL10*, and *PPARG*) in (**A**) HT29 cells and (**B**) SW620 cells determined by real-time PCR. The measurements were made with genistein 100 μM or 0.1% DMSO (control cells) treatment for 48 h. Values are expressed as mean ± SEM and control values (DMSO-treated cells) were set at 1.00. The coordinate values represent mRNA levels (arbitrary units). GEN, genistein; A.u., arbitrary units. * Significant difference between control cells and genistein-treated cells (Student’s *t*-test; *p* < 0.05, *n* = 6).

To understand the changes in inflammation, the mRNA expression levels of different interleukins and their receptors, as well as key genes of different inflammation-related pathways, were studied after 48 h of GEN 100 µM treatment. The HT29 cell line (Figure 8A) presented a statistically significant increase in *TNF, IL1B, CXCR2, HPSE*, and *IL10* (+594%, +97%, +135%, +115%, and +21%, respectively) and a statistically significant decrease in *CXCL8* (−19%) mRNA expression after GEN treatment. On the other hand, SW620 cells (Figure 8B) suffered a statistically significant increase in *TNF, CXCL8, CXCR2*, and *HPSE* (+814%, +174%, +2539%, and +676%, respectively) and a statistically significant decrease in *PPARG* (−70%) mRNA expression after GEN treatment. *IL1B* in SW620 cells also suffered an increase since the control cells showed a 40.6 ± 3.1 crossing point and GEN-treated cells showed a 31.9 ± 0.2 crossing point (data not shown).

### 2.8. GEN Affected NF-κB Translocation into the Nucleus

**Figure 9:**
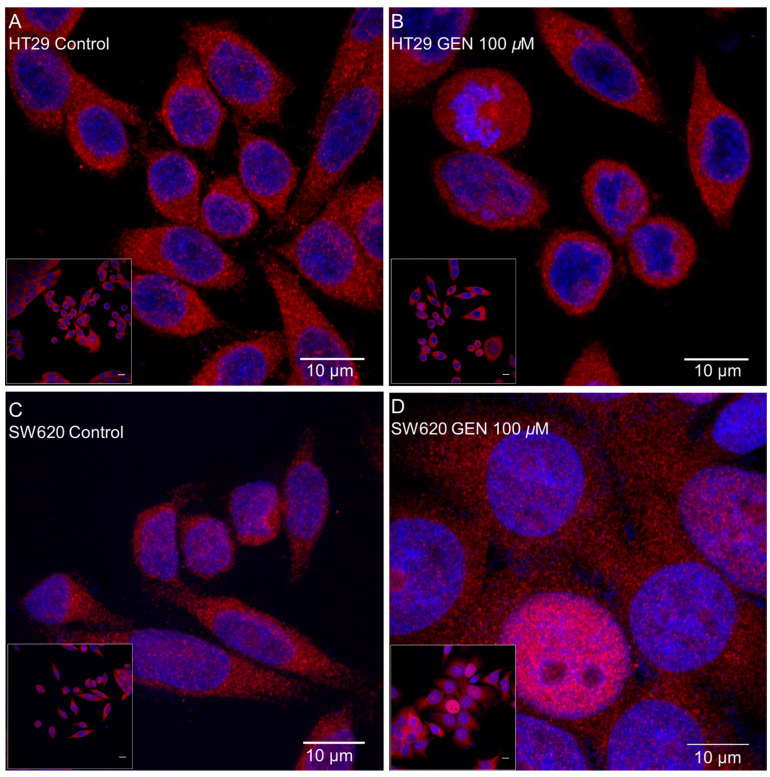
Effects of genistein on NF-κB nuclear translocation in (**A**,**B**) HT29 cells and (**C**,**D**) SW620 cells determined by immunocytochemistry. The measurements were made with 0.1% DMSO (control cells, (**A**,**C**)) or genistein 100 μM (**B**,**D**) treatment for 48 h. The fluorescence was monitored with a Leica TCS-SPE Confocal Microscope, using 63× immersion oil (147 N.A.) objective lens. Scale bar 10 μm. White square in the bottom left corner shows the whole field without zoom and the image in the center shows a zoom from the white square image. Nucleus and NF-κB proteins are represented in the picture in blue and red, respectively. The merge between both (pink) indicates that NF-κB had translocated into the nucleus.

Inflammatory-related gene changes were more pronounced in the SW620 cell line than in the HT29 cell line, thus, in order to have a more functional parameter related to inflammation, NF-κB translocation to the nucleus was studied after 48 h of GEN 100 µM treatment. Figure 9 displays confocal microscopy pictures taken after anti-NF-κB primary antibody and Hoechst 33342 (for the nucleus staining) incubation, where an increase of NF-κB translocation into the nucleus after GEN treatment in both cell lines can be observed, but it is more pronounced in the SW620 cells (Figure 9D), which is represented as the merge (pink) of the nucleus (blue) and NF-κB proteins (red). Furthermore, an increase of the SW620 cells’ size can be seen in Figure 9D due to an increase in swelling.

## 3. Discussion

The effects of high concentrations of the phytoestrogen GEN (100 µM) in colon cancer cells were analyzed to see the action of this phytoestrogen in cell viability, oxidative stress, mitochondrial biogenesis, and inflammation in colorectal cancer.

Some studies about GEN metabolization and digestion suggest that it could be accumulated in various colon areas, where GEN may exert its effects [26]. For this reason, a high intake of GEN-rich food can cause high doses of GEN to be, physiologically, in some colon areas and, therefore, it could be an interesting point to be studied. The decrease in cell viability with high GEN concentrations we found, which was more pronounced in SW620 cells, was also seen by Lepri et al., who observed an inhibition of cell viability in HT29 colon cancer cells treated with GEN 50 µM and 100 μM [27]. Furthermore, Xiao and collaborators observed an inhibition of cell growth in HT29 and SW620 cells after GEN treatment in a dose- and time-dependent manner [28].

This diminution in cell viability could be related to changes in cell cycle regulation. In HT29 cells, the decrease in cell viability could be promoted by the increase in cell death observed in the cell cycle analysis. The increase in the sub G_0_/G_1_ phase was previously seen by Salti and collaborators in the HT29 cells with a treatment with GEN 100 µM for 72 h [29]. In contrast, SW620 cells presented a G_2_/M cell cycle arrest of more than 85% of the cells, which could explain the decrease in cell viability due to the inability of SW620 cells to divide, causing a cytostatic effect of GEN on this cell line [30].

The results obtained in the cell viability and cell cycle analysis could be linked to an increase in H_2_O_2_ production [31]. HT29 and SW620 showed an increase in both *SOD2/CAT* and *SOD2/GPX1* mRNA expression ratios. Moreover, SW620 cells showed an increase in both the MnSOD/Catalase and MnSOD/GPx protein expression ratios, which results in a more pronounced increase in H_2_O_2_ accumulation in this cell line [17,18]. In fact, the accentuated rise in H_2_O_2_ production in SW620 cells and, consequently, in oxidative stress, results in the pronounced decrease in cell viability at higher GEN concentrations. This increase in oxidative stress was also reported by other authors after GEN 50 μM and 100 μM treatment in breast and colon cancer cells [32,33].

The different effects of GEN in the cell cycle according to the cell line, being more cytotoxic on HT29 cells and more cytostatic on SW620 cells, could be responsible for the difference in cell viability and ROS levels. The HT29 cells suffered cell death, and for this reason, the increase in the ROS levels is lower than in the SW620 cell line. In the SW620 cells, the increase in the ROS levels could be responsible for the cell cycle arrest [34,35] and the subsequent decrease in cell viability.

Stress fibers, the most relevant contractile structures in cells, participate in cellular–cellular and cellular–matrix union formations, cytokinesis, and migration [36]. In addition, filopodia are important for slow migration and can act as sensors and guide organelles [37]. Different studies showed that an increase of H_2_O_2_ production reorganizes the actin filaments producing stress fibers, filopodia, and lamellipodia in different cell lines, such as HAEC cells (human aortic endothelial cells) [38], SVEC4-10 cells (mouse endothelial cells) [39], and Rat2 fibroblasts [40]. The increase in the number of stress fibers in HT29 cells and filopodia in SW620 cells could be related to the increase in H_2_O_2_ production and the inflammatory status. In addition to this, the increase in stress fibers and filopodia in HT29 and SW620 cells could be related to the increase in the inflammatory state, since Costanzo and collaborators observed an increase in the number of stress fibers in CaCo2 and HT29 cell lines after TNFα and IFNγ treatment [41]. Finally, Cui and collaborators demonstrated that RAW 264.7 cells (a murine macrophage cell line), after GEN 50–100 µM treatment for 24 or 48 h, developed long pseudopodia-like protrusions [42].

In the HT29 cell line, mitochondrial biogenesis would be increased after GEN treatment, since after 48 h of treatment, *PPARGC1α* mRNA levels were lower and *TFAM* and *SSBP1* mRNA levels were higher. PGC1α, the most upstream mitochondrial biogenesis regulator with the highest activation in the short term [43] implicated in mtDNA transcription [44], promotes TFAM transcription [43] which, in turn, promotes mtDNA transcription [45], and mtSSB participates in mtDNA replication [46]. The new mitochondria could smooth the increase in H_2_O_2_ caused by GEN 100 µM treatment, leading to a slight accumulation of H_2_O_2_. Conversely, SW620 cells had a decrease in the mRNA expression levels of *ESRRA*, *TFAM*, and *SSBP1*, in addition to a decrease in mtDNA expression levels, which could imply a decrease in mitochondrial biogenesis. Furthermore, a recent study has demonstrated that silencing TFAM increases the H_2_O_2_ intracellular levels in SW620 cells and compromises SW620 viability due to its high energy demand [47]. All the data suggest that the decrease in the mRNA expression levels of mitochondrial biogenesis-related genes in SW620 cells could lead to a lack of mitochondrial renewal, so a great number of non-functional mitochondria could be accumulated, leading to a strong accumulation of H_2_O_2_ and, subsequently, a decrease in cell viability and cell cycle arrest.

The SW620 cell line showed an increase in inflammation, which can be seen by the increase in the pro-inflammatory genes’ expression and the decrease in the anti-inflammatory genes’ expression. Moreover, the increase in the NF-κB translocation to the nucleus demonstrated that this protein is more activated [48]. This cell line also presented swelling (Figure 9D) after GEN treatment, that could corroborate the G_2_/M arrest. The increase in inflammation is caused by the accumulation of H_2_O_2_, which activates NF-κB, allowing its translocation to the nucleus, where it can act as a transcription factor, modulating proinflammatory-related genes’ expression [23]. The decrease of *PPARG* also allows the translocation of NF-κB to the nucleus, since PPARγ inactivates NF-κB by avoiding the nuclear factor kappa B inhibitor (IκB) phosphorylation and retaining NF-κB in the cytoplasm [49]. Inside the nucleus, NF-κB permits the transcription of several cytokines (IL-1β, IL-8) and TNFα [23,49], increasing the inflammatory status of the cell. The increase in *TNF* produces an increase in *HPSE*, the main function of heparanase is the degradation of the extracellular matrix, releasing proinflammatory cytokines that are inside the matrix [50], and the transcription of TNFα and IL-1β [51]. Altogether, positive feedback is created and allows the perpetuation of the inflammatory status in SW620 cells. This inflammatory status, initially caused by the increase of H_2_O_2_, which cannot be palliated neither by the antioxidant enzymes nor mitochondrial biogenesis, could lead SW620 cells to G_2_/M cycle arrest and, consequently, a decrease in cell viability. In the HT29 cell line, there was an increase in the inflammatory status that, in comparison, is less pronounced than in the SW620 cell line. HT29 cells suffered a less pronounced increase in proinflammatory genes than SW620 cells, which could be due to the minor accumulation of H_2_O_2_ in these cells, and furthermore, an increase in the anti-inflammatory gene *IL10*’s expression, which can be increased in order to palliate and produce lower levels of inflammation [52]. Moreover, despite the lower levels of H_2_O_2_ production compared to SW620 cells, NF-κB translocation to the nucleus was observed, but this translocation was less pronounced than in the SW620 cell line. Previous studies demonstrate that high concentrations of GEN in HT29 cells inhibit NF-κB translocation to the nucleus [53,54], since GEN has antioxidant and anti-inflammatory properties, but our results demonstrate that a high concentration of GEN treatment has pro-oxidant and pro-inflammatory properties. In this cell line, increased apoptosis produced a lower inflammatory status, caused by the lower levels of H_2_O_2_ and the increase of *IL10.*

## 4. Materials and Methods

### 4.1. Reagents

Dulbecco’s Modified Eagle’s Medium (DMEM) high glucose was purchased from GIBCO (Paisley, UK). Fetal bovine serum and penicillin–streptomycin solution were purchased from Biological Industries (Kibbutz Beit Haemek, Israel). GEN was obtained from Sigma-Aldrich (St-Louis, MO, USA). Routine chemicals were supplied by Panreac (Barcelona, Spain), Sigma-Aldrich (St Louis, MO, USA), Bio-Rad Laboratories (Hercules, CA, USA), and Roche (Barcelona, Spain).

### 4.2. Cell Culture

Human colon cancer cell lines HT29 and SW620 were obtained from American Type Culture Collection (ATCC; HT-29 (ATCC HTB-38) and SW-620 [SW-620] (ATCC CCL-227); Manassas, VA, USA), and cultured in DMEM supplemented with 10% heat-inactivated fetal bovine serum (FBS) (*v*/*v*) and 1% penicillin and streptomycin (*v*/*v*) at 37 °C with 5% CO_2_.

Cells were seeded at similar passages and treated, the following day, at 70% of confluency with increasing concentrations (1, 5, 50, and 100 μM) of GEN with 0.1% dimethyl sulfoxide (DMSO) as the vehicle, and the control vehicle-cells were treated with 0.1% DMSO for 48 h for cell viability and hydrogen peroxide (H_2_O_2_) production determinations. For further experiments, both cell lines were treated only with 100 μM of GEN for 48 h, and control vehicle-treated cells were treated with 0.1% DMSO.

### 4.3. Cell Viability Determination

Total of 5 × 10^4^ HT29 cells and 4 × 10^4^ SW620 cells were seeded in each well in a 96-well plate and treated with increasing concentrations of GEN for 48 h. Cell viability was determined with Hoechst 33342 (Sigma-Aldrich, St Louis, MO, USA), which emits blue fluorescence when it is bound to double strand DNA. After GEN treatment, the culture medium was removed, and 5 μg/mL of Hoechst was incubated for 5 min at 37 °C. Finally, FLx800 microplate fluorescence reader (BIO-TEK, Winooski, VT, USA) was used to measure the fluorescence, set at excitation wavelength of 350 nm and emission wavelength of 455 nm.

### 4.4. Fluorimetric Determination of H_2_O_2_ Production

H_2_O_2_ production was determined using an Amplex^®^ Red Hydrogen Peroxide/Peroxidase Assay Kit (A22188, Fisher Scientific, Madrid, Spain), according to the method described by Pons et al. [55]. Total of 5 × 10^4^ HT29 cells and 4 × 10^4^ SW620 cells were seeded in each well in a 96-well plate and treated with increasing concentrations of GEN for 48 h. FLx800 microplate fluorescence reader (BIO-TEK, Winooski, VT, USA) was used to measure fluorescence, set at excitation wavelength of 570 nm and emission wavelength of 585 nm. The maximum slope of the increase in the fluorescence was detected within 30 min of exposure to kit reagents. The number of viable cells determined by Hoechst 33342, as previously described, was used to normalize the obtained values.

### 4.5. RNA Isolation, RT-PCR, and Real-Time PCR

Total of 1.5 × 10^6^ HT29 cells and 2.1 × 10^6^ SW620 cells were seeded in each well in 6-well plates and treated with GEN 100 µM for 48 h. Then, total RNA was isolated by using Tri Reagent^®^ (Catalog no. T9424, Sigma-Aldrich) following the manufacturer’s protocol. A BioSpec-nano spectrophotometer (Shimadzu Biotech, Kyoto, Japan) was used to quantify the total RNA amount, set at wavelength of 260 nm. The RNA quality was checked by 260/280 and 260/230 ratios.

Then, 1 μg of the total RNA was reverse transcribed to cDNA, according to Pons et al., [55]. cDNA aliquots were frozen after 1/10 dilution in free-RNAase water (−20 °C).

A LightCycler 480 System II rapid thermal cycler (Roche Diagnostics, Basel, Switzerland) with SYBR Green technology was used to carry out the real-time PCR. The expression of copper–zinc superoxide dismutase (*SOD1*), manganese superoxide dismutase (*SOD2*), glutathione peroxidase (*GPX1*), catalase (*CAT*), peroxisome proliferator-activated receptor gamma coactivator 1-alpha (*PPARGC1α*), estrogen-related receptor alpha (*ESRRA*), transcription factor A (*TFAM*), single-stranded mitochondrial binding protein 1 (*SSBP1*), tumor necrosis factor alpha (*TNF*), interleukin-1 beta (*IL1B*), interleukin-8 (*CXCL8*), interlukin-8 receptor (*CXCR2*), heparanase (*HPSE*), interlukin-10 (*IL10*), and peroxisome proliferator-activated receptor gamma (*PPARG*) genes were analyzed, using the expression of beta-2-microglobulin (*B2M*) as housekeeping gene. Genes, primers, and annealing temperatures used are shown in Table 1.

The total reaction volume was processed according to Pons et al. [55], with SYBR green TB Green Premix ExTaq (TAKARA, RR420A). The first step in the amplification program was a preincubation to achieve the denaturation of the template cDNA (5 min, 95 °C), then 40 cycles of denaturation (10 s, 95 °C), followed by annealing (10 s, primer-specific temperature, shown in Table 1) and elongation (12 s, 72 °C). For each gene, a negative control without cDNA was loaded in the real-time PCR.

To analyze the Cp values of the real-time PCR, GenEx Standard Software (Multi-DAnalises, Göteborg, Sweden) was used considering the efficiency of the reaction for each pair of primers and normalizing with the *B2M* housekeeping gene.

### 4.6. DNA Isolation and Real-Time PCR

Total of 1.5 × 10^6^ HT29 cells and 2.1 × 10^6^ SW620 cells were seeded in 6-well plates and treated with GEN 100 µM for 48 h. Then, DNA was isolated by using Tri Reagent^®^ (Catalog no. T9424, Sigma-Aldrich), following the manufacturer’s protocol. A BIO-TEK PowerWave XS spectrophotometer was used to quantify the DNA amount, set at wavelength of 260 nm. The DNA quality was checked by 260/280 ratio. A total amount of 5 ng of DNA was carried out into a real-time PCR, as previously described, and the expression of mitochondrial DNA (mtDNA) was analyzed, using the expression of *18S* as housekeeping gene. Genes, primers, and annealing temperatures used are shown in Table 1.

### 4.7. Western Blot Analysis

Total of 8 × 10^5^ HT29 cells and 8 × 10^5^ SW620 cells were seeded in each well in 6-well plates and treated with GEN 100 µM for 48 h. Cells were harvested in PBS (137 mM NaCl, 2.7 mM KCl, 10 mM Na_2_HPO_4_, 2 mM KH_2_PO_4_, pH 7.4) with a scraper, and centrifugated at 600 g for 5 min. The pellet was dissolved in RIPA buffer (50 mM Tris-HCl, pH 7.5, 150 mM NaCl, 0.1% SDS, 0.5% deoxycholate, 1% Triton X-100, 1 mM EDTA) with protease inhibitors (Halt protease and phosphatase inhibitor single-use cocktail, EDTA-free 100X, Thermo Scientific 78443) in a proportion of 100:1. Then, cells were sonicated with Vibra Cell Ultrasonic Processor 75185 on ice in three cycles of 25W for 10 s with an interval of 5 s between each pulse and 40% of amplitude. After that, cells were centrifugated at 600 g for 5 min. The supernatant was recovered and the protein amount was quantified by the BCA method (Thermo Scientific™ 23227), following the manufacturer’s protocol.

For all SDS-PAGE carried out, 20 µg of total protein were loaded in each well. Glyceraldehyde-3-phosphate dehydrogenase (GAPDH) was used as loading control. Proteins were separated by electrophoresis on 12% acrylamide/bisacrylamide (30/1) gel, after that, proteins were electrotransferred, by semi-dry electrotransfer, on a 0.2 µm nitrocellulose membrane (Bio-Rad Laboratories, Hercules, CA, USA) using the Trans-blot Turbo transfer system (Bio-Rad Laboratories, CA, USA). Then, membranes were blocked with 5% non-fat powdered milk in TBS-Tween (Tris Buffer Saline Tween, pH 7.6 containing 0.05% Tween-20) for 1 h at room temperature and agitation. After that, membranes were incubated over night at 4 °C in agitation with primary antibody (5% bovine serum albumin and 0.05% sodium azide in TBS-Tween). The primary antibodies used and their respective dilutions were: copper–zinc superoxide dismutase (CuZnSOD) 1:1000 (Calbiochem, 574597), manganese superoxide dismutase (MnSOD) 1:500 (Santa Cruz, 30080), gluthatione peroxidase (GPx) 1:500 (Santa Cruz, 133160), catalase 1:1000 (Calbiochem, 219010), and GAPDH 1:1000 (Santa Cruz, 365062). Finally, membranes were incubated with horseradish peroxidase-conjugated secondary antibody (2% non-fat powdered milk in TBS-Tween) for 1 h at room temperature and agitation. The secondary antibodies used, and their respective dilutions, were: anti-rabbit 1:10,000 (Sigma, A9169), anti-mouse 1:10,000 and 1:2000 for GPx (Sigma, A9044), and anti-sheep 1:10,000 (Sigma, A3415). To detect the immunoreactivity, Inmun-Star© Western Chemilumeniscence kit Western blotting detection systems (Bio-Rad Laboratories, Hercules, CA, USA) were used. Chemiluminescent signal was acquired with Chemidoc XRS densitometer (Bio-Rad Laboratories, Hercules, CA, USA), and the results were analyzed with Quantity One Software (Bio-Rad Laboratories, Hercules, CA, USA).

### 4.8. Cell Cycle Analysis

Cell cycle analysis was done by flow cytometry. Total of 9 × 10^5^ HT29 cells and 8 × 10^5^ SW620 cells were seeded in each well in 6-well plates and treated with GEN 100 µM for 48 h. After that, cells were harvested with trypsin-EDTA and fixed in cold 100% methanol. Fixed cells were incubated at −20 °C overnight and centrifugated for 5 min at 600× *g*. Before the analysis for DNA staining, cells were incubated at room temperature in the dark for 30 min with an RNAase and propidium iodide mix. Flow cytometry experiments were performed using a Beckton-Dickinson FACSVerse flow cytometer, and the results were analyzed with FACSuite v1.0.6 software.

### 4.9. Immunocytofluorescence with Confocal Microscopy

Total of 5 × 10^5^ HT29 cells and 5 × 10^5^ SW620 cells were seeded on a glass coverslip inside 6-well plates and treated with GEN 100 µM for 48 h. Cells were washed with PBS-T (0.1% Tween 20) and then were fixed with 4% paraformaldehyde (Panreac, 141451.1210) in PBS (pH 7.4) for 10 min at room temperature. After that, cells were washed with cold PBS. Permeabilization was carried out with 0.25% Triton X-100 (Sigma, X-100) in PBS for 10 min at room temperature, and then cells were washed with PBS. Then, cells were blocked with 1% BSA (Sigma, A4503-50G) with 22.52 mg/mL glycine (Panreac, A1067) in PBS-T for 30 min at room temperature. After blocking, cells were incubated with NF-κB primary antibody 1:50 (Santa Cruz, sc-372) with 1% BSA in PBS-T during 1 h in a humidified chamber at room temperature. After incubation, cells were washed with PBS and incubated with anti-rabbit secondary antibody 1:100 (Alexa fluor 555, A-21429, Invitrogen) with 1% BSA in PBS-T for 1 h in a humidified chamber at room temperature in the dark. Before the DNA staining, cells were washed with PBS. For the DNA staining, cells were incubated with 1 μg/mL Hoechst 33342 (Sigma, B2261) in PBS for 1 min at room temperature in the dark and then washed with PBS. Finally, coverslip was mounted with a drop of DAKO Fluorescent Mounting Medium (DAKO, S3023) and incubated O/N at room temperature in the dark.

The fluorescence was monitored with a Leica TCS-SPE Confocal Microscope, using 63× immersion oil (147 N.A.) objective lens. Fluorescence emission was 555 nm.

### 4.10. Actin Cytoskeleton Remodeling Determination with Confocal Microscopy

Total of 5 × 10^5^ HT29 cells and 5 × 10^5^ SW620 cells were seeded on a glass coverslip inside 6-well plates and treated with GEN 100 µM for 48 h. Cells were washed with PBS and then were fixed with 4% paraformaldehyde in PBS (pH 7.4) for 10 min at room temperature. After that, cells were washed with PBS and were stained with 0.1 mg/mL Phalloidin–Tetramethylrhodamine B isothiocyanate (1:1000, Sigma, P1951) for 1 h at 37 °C. After staining, cells were washed with PBS and, for the DNA staining, cells were incubated with 10 mg/mL DAPI (1:1000, Sigma, D9542) for 10 min at room temperature, and then washed with PBS. Finally, coverslip was mounted with a drop of DAKO Fluorescent Mounting Medium and incubated O/N at room temperature in the dark.

The fluorescence was monitored with a Leica TCS-SPE Confocal Microscope, using 63× immersion oil (147 N.A.) objective lens. Fluorescence emission wavelength was 570 nm and fluorescence excitation wavelength was 532 nm.

### 4.11. Statistical Analysis

Statistical Program for the Social Sciences software for Windows (SPSS, version 24.0; SPSS Inc., Chicago, IL, USA) was used to perform all the statistical analyses. Student’s t-test was used to analyze the differences between control and treated cells, with minimal statistical significance at *p* < 0.05. All results are presented as mean values (*n* = 6) ± standard error of the mean (SEM).

## 5. Conclusions

High concentrations of genistein induced a decrease in cell viability in colon cancer cells that could be promoted by changes in cell cycle regulation. In colon primary cancer HT29 cells, the increase in cell death promotes a slight raise of oxidative stress that can be mitigated by the increase in mitochondrial biogenesis; the increase in ROS production increments the inflammatory status, and finally, results in a moderate decrease in cell viability. On the other hand, in colon metastatic cancer SW620 cells, the significant increase in ROS production, that cannot be palliated by antioxidant enzymes nor mitochondrial biogenesis, produces a great rise in inflammatory levels, that all together results in a G_2_/M cell cycle arrest and a decrease in cell viability. Although further studies are necessary to better understand the role of genistein during anticancer therapy, this work provides new insights into the effects of high doses of this phytoestrogen on the physiology of colon cancer cells.

## Figures and Tables

**Table 1:** Primers sequences and their respective annealing temperatures and accession number.

Gene	Forward Primer (5′-3′) Reverse Primer (5′-3′)	Annealing Temperature (°C)	Accession Number
*B2M*	5′-TTT CAT CCA TCC gAC ATT GA-3′ 5′-Cgg CAg gCA TAC TCA TCT TT-3′	54	NM_004048
*SOD2*	5′-CgT gCT CCC ACA CAT CAA TC-3′ 5′-TgA ACg TCA CCg Agg AgA Ag-3′	64	BT006967
*SOD1*	5′-TCA ggA gAC CAT TgC ATC ATT-3′ 5′-CgC TTT CCT gTC TTT gTA CTT TCT TC-3′	64	NM_000454
*CAT*	5′-CAT CgC CAC ATg AAT ggA TA-3′ 5′-CCA ACT ggg ATg AgA ggg TA-3′	61	NM_001752
*GPX1*	5′-gCg gCg gCC Cag TCg gTg TA-3′ 5′-gAg CTT ggg gTC ggT CAT AA-3′	61	M21304
*PPARGC1A*	5′-TCA gTC CTC ACT ggT ggA CA-3′ 5′-TgC TTC gTC gTC AAA AAC Ag-3′	60	AF106698
*ESRRA*	5′-TCg CTC CTC CTC TCA TCA TT-3′ 5′-Tgg CCA AAC CCA AAA ATA AA-3′	52	NM_004451
*TFAM*	5′-gTg gTT TTC ATC TgT CTT ggC-3′ 5′-ACT CCg CCC TAT AAg CAT CTT-3′	60	BT019658
*SSBP1*	5′-TgT gAA AAA ggg gTC TCg AA-3′ 5′-Tgg CCA AAg AAg AAT CAT CC-3′	60	AF277319
*TNF*	5′-AAg CCT gTA gCC CAT gTT gT-3′ 5′-ggA CCT ggg AgT AgA TgA ggT-3′	58	NM_000594
*IL1B*	5′-TCg CCA gTg AAA TgA Tgg CT-3′ 5′-ggT Cgg AgA TTC gTA gCT gg-3′	58	BT007213
*CXCL8*	5′-ggC ACA AAC TTT CAg AgA CAg CAg-3′ 5′-gTT TCT TCC Tgg CTC TTg TCC TAg-3′	66	AK311874
*CXCR2*	5′-AgT TCT Tgg CAC gTC ATC gT-3′ 5′-CCC CTg AAg ACA CCA gTT CC-3′	57	M68932
*HPSE*	5′-gCA AAC TgC TCA ggA CTg gA-3′ 5′-gCT gAC CAA CAT CAg gAC CA-3′	60	AF084467
*IL10*	5′-ACA TCA Agg CgC ATg TgA AC-3′ 5′-CAC ggC CTT gCT CTT gTT TTC-3′	60	M57627
*PPARG*	5′-gAg CCC AAg TTT gAg TTT gC-3′ 5′-CTg TgA ggA CTC Agg gTg gT-3′	61	BT007281
*mtDNA*	5′-CgT gAC TCC TAC CCC TCA CA-3′ 5′-ATC ggg TgA TAg CCA Ag-3′	60	NM_025230.5
*18S*	5′-ggA CAC ggA CAg gAT TgA CA-3′ 5′-ACC CAC ggA ATC gAg AAAGA	60	NR_146119.1

## Data Availability

Not applicable.
